# Drugs against metabolic diseases as potential senotherapeutics for aging-related respiratory diseases

**DOI:** 10.3389/fendo.2023.1079626

**Published:** 2023-04-03

**Authors:** Sachi Matsubayashi, Saburo Ito, Jun Araya, Kazuyoshi Kuwano

**Affiliations:** Division of Respiratory Diseases, Department of Internal Medicine, The Jikei University School of Medicine, Tokyo, Japan

**Keywords:** senotherapy, senolytics, senomorphics, drugs against metabolic diseases, aging-related respiratory disease, chronic obstructive pulmonary disease (COPD), idiopathic pulmonary fibrosis (IPF), inhalation therapy

## Abstract

Recent advances in aging research have provided novel insights for the development of senotherapy, which utilizes cellular senescence as a therapeutic target. Cellular senescence is involved in the pathogenesis of various chronic diseases, including metabolic and respiratory diseases. Senotherapy is a potential therapeutic strategy for aging-related pathologies. Senotherapy can be classified into senolytics (induce cell death in senescent cells) and senomorphics (ameliorate the adverse effects of senescent cells represented by the senescence-associated secretory phenotype). Although the precise mechanism has not been elucidated, various drugs against metabolic diseases may function as senotherapeutics, which has piqued the interest of the scientific community. Cellular senescence is involved in the pathogenesis of chronic obstructive pulmonary disease (COPD) and idiopathic pulmonary fibrosis (IPF), which are aging-related respiratory diseases. Large-scale observational studies have reported that several drugs, such as metformin and statins, may ameliorate the progression of COPD and IPF. Recent studies have reported that drugs against metabolic diseases may exert a pharmacological effect on aging-related respiratory diseases that can be different from their original effect on metabolic diseases. However, high non-physiological concentrations are needed to determine the efficacy of these drugs under experimental conditions. Inhalation therapy may increase the local concentration of drugs in the lungs without exerting systemic adverse effects. Thus, the clinical application of drugs against metabolic diseases, especially through an inhalation treatment modality, can be a novel therapeutic approach for aging-related respiratory diseases. This review summarizes and discusses accumulating evidence on the mechanisms of aging, as well as on cellular senescence and senotherapeutics, including drugs against metabolic diseases. We propose a developmental strategy for a senotherapeutic approach for aging-related respiratory diseases with a special focus on COPD and IPF.

## Introduction

The global population is aging at an unprecedented rate. Aging adversely affects physiological functions and consequently increases the susceptibility of most organs to various pathological conditions. In the respiratory system, aging can induce pathological changes, such as deterioration of respiratory function, increased susceptibility to infection, and malignancy (1, 2). The incidence of chronic obstructive pulmonary disease (COPD) and idiopathic pulmonary fibrosis (IPF), which are representative aging-related respiratory diseases, increases with age (3). COPD and IPF are a major socioeconomic burden due to the cumulative cost and effort associated with medical events. Hence, there is an urgent need to understand the pathogenesis of aging-related respiratory diseases and develop novel therapies. In recent years, basic research on aging mechanisms has markedly advanced. The identification of the lifespan-extending effect of caloric restriction in model organisms and the subsequent discovery of lifespan-related genes (represented by Sirtuin-encoding genes) provide clues for understanding the molecular mechanisms of aging (4–7). Furthermore, mouse studies involving genetic manipulation of cyclin-dependent kinase (CDK) inhibitory proteins, which regulate the cell cycle, have demonstrated that the elimination of senescent cells can extend lifespan and mitigate the development of various aging-related diseases (8, 9). Thus, aging research is now rapidly developing.

In addition to their effects on the primary target diseases, drugs against metabolic diseases, such as diabetes and dyslipidemia, may exert therapeutic effects on various aging-related diseases (10). Metabolic diseases are common complications in patients with aging-related respiratory diseases. Hence, the anti-aging effects of drugs against metabolic diseases are attracting attention in the field of respiratory diseases. Retrospective studies on metabolic disorders have delineated several interesting findings. For example, statins are reported to reduce the incidence of COPD exacerbation episodes and mitigate the decline in respiratory function (11, 12). The anti-diabetic drug metformin is also expected to suppress decline of lung function in COPD patients (13). In patients with both COPD and type 2 diabetes, the mortality rate among metformin users is lower than that of the control cohort with similar backgrounds (14). Further to attributing their therapeutic effects on metabolic diseases, recent advances indicate that these drugs may directly suppress cellular senescence.

Cellular senescence is phenotypically characterized by irreversible cell cycle arrest and apoptosis resistance, which are adaptive responses to various intrinsic and extrinsic stresses (15, 16). Senescent cells are also characterized by a senescence-associated secretory phenotype (SASP) that produces various cytokines and growth factors. Excessive SASP caused by the accumulation of senescent cells has been implicated in chronic inflammation, aberrant tissue repair, and fibrotic tissue remodeling. Therefore, the regulation of cellular senescence is proposed to be a promising approach to prevent aging-related diseases. Many researchers are focusing on an anti-senescence modality of treatment, namely senotherapy (17). Although various new agents are being developed, repositioning pre-existing drugs with potential clinical efficacy as a senotherapeutic can be a reasonable strategy. If drug repositioning, which is also called repurposing, is possible without safety concerns, it will enable the immediate clinical application of the drug. In this review, we discuss the repositioning of drugs against metabolic diseases as potential senotherapeutics for aging-related respiratory diseases with a special focus on COPD and IPF.

## Mechanism of aging

Organisms employ dynamic defense mechanisms, which are called homeostasis and robustness, to maintain stability against internal and external disturbances. Homeostasis refers to short-term mechanisms, whereas robustness refers to systemic long-term protective mechanisms, including defense mechanisms against perturbation, dynamic responses to environmental changes, and tissue regeneration in response to injury or defect (18–20). Organismal aging can be, at least partly, assumed to be the dysregulation of robustness (21). Therefore, the organism becomes fragile, exhibiting a reduced ability to respond to changes and insults and a low ability to regenerate. Aging-induced impaired robustness, which is associated with the accumulation of senescent cells, is believed to result from a decline in organ function. The hallmarks of aging include genomic instability, telomere attrition, epigenetic alterations, loss of proteostasis, disabled macroautophagy, deregulated nutrient-sensing, mitochondrial dysfunction, cellular senescence, stem cell exhaustion, altered intercellular communication, chronic inflammation, and dysbiosis (22). These pathological changes are interdependent and can drive the progression of cellular senescence. Additionally, aging is a heterogenous process even at the cellular level. The aging process is not equal in all cells with some and not all cells exhibiting senescence (23, 24), indicating the presence of complex mechanisms for regulating organismal aging. To explore a druggable approach against this complex aging process, the molecular mechanisms of aging have been widely investigated. Recent studies have suggested that the accumulated senescent cells can be a promising therapeutic target. Senotherapeutics, which target cellular senescence, are classified into the following two types: senolytics, which induce cell death in senescent cells, and senomorphics, which suppress the SASP-inducing effect and prevent cellular senescence. In the following section, we provide an overview of cellular senescence and a recent understanding of senotherapy.

## Cellular senescence and concept of senotherapeutics (senolytics and senomorphics)

Cellular senescence, first reported by Hayflick, is a phenomenon that repeatedly divided human fibroblasts *in vitro* cannot proliferate beyond certain limits and have finite proliferative capacity (25). Initially, this phenomenon was attributed to an artificial change that occurs only under cell culture conditions. However, accumulated evidence revealed that cellular senescence has physiological roles (26–30) and is involving in aging (31–34). Internal and external stimuli, such as DNA damage stress (induced by radiation, chemotherapy, and reactive oxygen species (ROS)), inflammation, mechanical stress, repeated cell growth signals (growth factors and insulin-like growth factor-1 (IGF-1) signaling), metabolic aberrations, mitochondrial dysfunction, accumulation of unfolded proteins, certain oncogenes, and nuclear membrane dysfunction, can induce cellular senescence (15). These stimuli activate several signaling pathways and can converge on IGF-1/Akt/mammalian target of rapamycin (mTOR) signaling, which regulates transcription factor cascades, including the cell cycle inhibitors p16^INK4A^/RB and p53/p21^CIP1^, resulting in sustained irreversible cell cycle arrest (35). Cellular senescence plays physiological roles in diverse conditions and the number of senescent cells increases with aging. Excessive and disorganized senescent cells promote chronic inflammation and fibrotic tissue remodeling, exert paracrine effects on distant organs, and consequently drive the pathogenesis of many aging-related diseases (16, 36).

Novel treatment strategies targeting cellular senescence have been investigated based on these molecular mechanisms. Senolytics have been validated using mouse models. Mice have been genetically engineered to selectively eliminate p16-expressing senescent cells. These mice exhibit enhanced lifespan and delayed onset of aging-related pathologies (8). Senolytics have been explored using the STRING database (functional protein association networks) based on the biological differences between healthy and senescent cells. Dasatinib (a pan-tyrosine kinase inhibitor originally developed as an anti-cancer drug) and quercetin (a flavonoid) were selected as novel senolytic drugs (37). Subsequently, the Bcl-2 family inhibitor navitoclax (ABT-263) and selective Bcl-xL inhibitors A1331852 and A1155463 have been developed (38, 39). Fisetin, a polyphenol, exhibits senolytic activity by inhibiting the phosphatidylinositol-3 kinase-mTOR pathway (38). The administration of these senolytic agents decreases the accumulation of senescent cells and inflammatory cytokines, improves physical activity, and enhances the lifespan in aged mice (40). Other compounds, such as cardiac glycosides (ouabain) and HSP90 inhibitors (geldanamycin derivatives) also exhibit senolytic activity (41, 42).

Senomorphics attenuate the pathological effect of SASP without inducing cell death. Rapamycin, resveratrol, and metformin are representative senomorphics (43). Promising senomorphics include aspirin, NF-κB inhibitors, p38MAPK inhibitors, JAK/STAT inhibitors, ATM inhibitors, and statins (17). The molecular mechanisms of senomorphics are more complex than those of senolytics. Based on the complex mechanism and physiological role of cellular senescence, most senomorphics target SASP. Cellular senescence is initially induced in a small number of stressed cells and subsequently induced in neighboring and distant cells through the paracrine/autocrine effect of SASP. Hence, senomorphics targeting SASP may block the induction of cellular senescence at multiple sites (17). Senomorphics not only suppress senescence expansion by targeting the initial few senescent cells but also inhibit a vicious cycle that promotes further accumulation of senescent cells. Senotherapeutics can be promising therapeutic agents and may prevent the progression of aging-related pathology.

**Figure 1:**
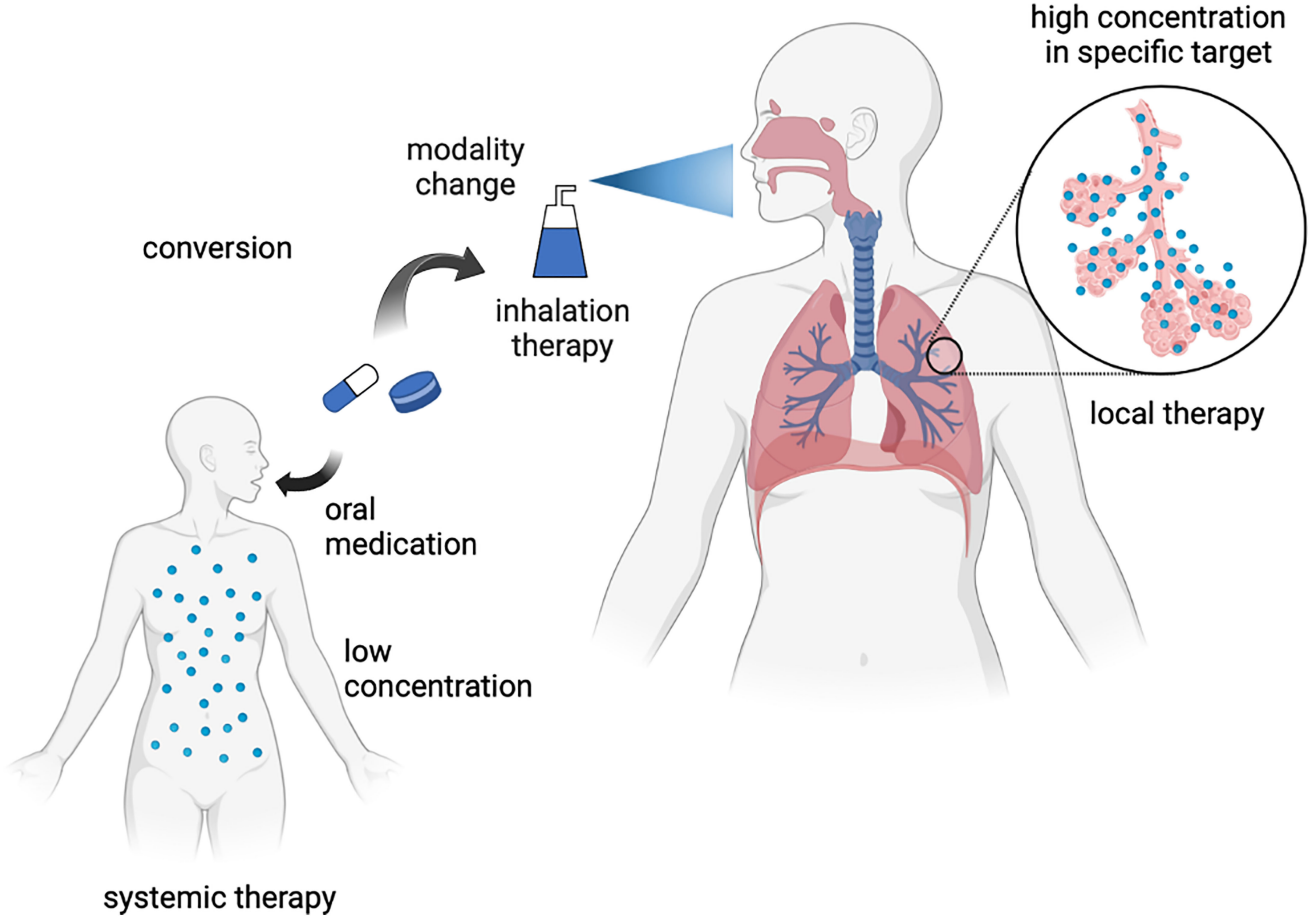
Concept of inhalation therapy with drugs against metabolic diseases for respiratory diseases as drug repositioning. Created with Biorender.

To develop a senotherapeutic strategy for aging-related respiratory diseases, one attractive approach is the repositioning of drugs as potential senomorphics. In contrast to *de novo* drug discovery, drug repositioning has several advantages, including low discovery costs and clinically established safety. A recent successful drug repositioning model is the application of an SGLT2 inhibitor, an anti-diabetic drug, for the treatment of heart failure (44, 45). In respiratory disease, another potential approach is changing the treatment modality to inhalation therapy, which may achieve a high local drug concentration in the lungs without exerting systemic adverse effects. Although several drugs may exert a senotherapeutic effect, high non-physiological concentrations are required under experimental conditions. Thus, the potential repositioning of drugs can be explored by changing the treatment modality to inhalation therapy for aging-related respiratory diseases (**Figure 1**). In the following section, we explain the involvement of cellular senescence in metabolic diseases and aging-related respiratory diseases and describe the potential therapeutic use of drugs against metabolic diseases as senotherapeutics.

## Metabolic diseases and cellular senescence

Cellular senescence is involved in the pathogenesis of various metabolic diseases (10), including metabolic syndrome, type 2 diabetes, and osteoporosis. Aging and obesity are the major risk factors for the development of type 2 diabetes (46). Senescent adipocytes accumulate in both mouse models and human cases of type 2 diabetes and obesity (47). Additionally, senescent adipocytes with p53 activation and enhanced ROS production are closely related to insulin resistance (48), glucose intolerance, and systemic inflammation (49), indicating the presence of a vicious cycle between adipocyte senescence and progression of type 2 diabetes. Furthermore, hyperglycemia and increased serum lipids induce adipocyte senescence and cellular senescence in various organs.

The efficacy of senolytics against metabolic diseases has been previously investigated. Senolytic therapy with dasatinib and quercetin (D + Q) is demonstrated to reduce the number of senescent adipocytes and restore insulin sensitivity in diet-induced obese mice (50). D + Q may also reduce the accumulation of senescent adipocytes in humans. The administration of D + Q for 3 days decreases the counts of macrophages and senescent adipocytes in adipose tissue and circulating SASP in patients with diabetic nephropathy (51). Thus, these results indicate that senolytics can be a promising approach for treating type 2 diabetes, which should be examined in future clinical trials. Additionally, the number of senescent cells is reported to increase with aging in the bone tissue in both mouse models and human samples (52, 53). D + Q, or the SASP inhibitor ruxolitinib alleviate osteoporosis in the mouse models (54).

Local organ aging may affect systemic aging in most metabolic diseases by inducing metabolic disturbances and upregulating SASP-related factors (10). Hence, the senotherapeutic effect of drugs against metabolic diseases is conferred through the improvement of metabolic conditions or the systemic anti-senescence properties during systemic administration. The therapeutic effect of drugs on metabolic diseases can be attributed to the accumulation of these pleiotropic effects. Moreover, this complex interaction between focal and systemic cellular senescence may be involved in other aging-related diseases. Several studies have demonstrated that drugs against metabolic disorders can exert a senotherapeutic effect on various cell types and tissues (targets other than the original therapeutic targets), including respiratory cells and tissues.

## Aging-related respiratory diseases and cellular senescence

### COPD and cellular senescence

Aging is closely associated with COPD development (55). Cellular senescence is observed in lung epithelial cells, vascular endothelial cells, and fibroblasts of patients with COPD (56, 57). Stimulation with cigarette smoke extract (CSE) induces senescence in the lung epithelial cells and lung fibroblasts *in vitro* (58–61). Additionally, cellular senescence and DNA damage in vascular endothelial progenitor cells and type II alveolar epithelial cells of patients with COPD are upregulated when compared with those cells of healthy controls, which may lead to the depletion of progenitor cells and stem cells required for regeneration of the damaged lung (62). In addition to increased numbers of senescent cells, elevated levels of SASP-related factors have been reported in the lungs of patients with COPD. In terms of COPD pathogenesis, the accumulation of senescent cells induced by repeated cigarette smoke exposure and chronological aging contribute to SASP-mediated inflammation, aberrant tissue repair, and loss of regenerative capacity, resulting in airway wall thickening and emphysema (3). Differential effects of cigarette smoke exposure on cellular senescence progression may be a critical determinant of COPD development. A recent study reported a negative correlation between epigenetic aging regulated by DNA methylation and respiratory function, suggesting a pivotal role of epigenetic modification in cellular senescence during COPD pathogenesis (63).

The expression of cellular senescence markers of CDK inhibitors (CDKI), such as p21^CIP1^ and p16^INK4A^, is upregulated in the airway epithelium of patients with COPD (58, 64). Several studies have demonstrated the potential efficacy of a senotherapeutic approach for preventing COPD development by regulating CDKI levels and CDKI-expressing cell numbers. Genetic deletion of p16^INK4A^ may suppress smoking-stimulated respiratory function decline, emphysema, and cellular senescence of the airway epithelium in mouse models (65). In contrast, other studies have reported that genetic deletion of p16^INK4A^ alone is not sufficient to suppress cellular senescence and emphysema (66). p14^ARF^ (p19^ARF^ in mice), encoded by the *INK4a-ARF* locus (also encodes p16^INK4A^; *CDKN2A* in humans), regulates cell cycle arrest and is used as a cellular senescence marker (67). The removal of p19^ARF^-expressing senescent cells by specifically inducing apoptosis with diphtheria toxin suppressed emphysematous changes induced by both cigarette smoke exposure and elastase in mouse models (68–70). Thus, the use of senolytics may be a promising approach for COPD; however, the safety and clinical application of senolytics for COPD treatment remain obscure. Moreover, the beneficial or adverse effects of a senolytic approach on organs with a high senescence burden, such as the lungs of patients with COPD have not been evaluated (71). A senomorphic approach using drugs against metabolic diseases for COPD is discussed in the following sections.

### IPF and cellular senescence

Aging is a known risk factor for IPF development. The incidence of IPF increases with age (72). Telomere shortening, mainly reflecting replicative cellular senescence, is reported to be closely involved in IPF pathogenesis. Mutations in the telomerase-encoding gene (*TERT*) are detected in 8%–15% of patients with familial pulmonary fibrosis (73, 74). The frequency of telomere shortening is high in patients with sporadic pulmonary fibrosis (75). A study on three large cohorts of patients with IPF reported that shorter telomere length is associated with poor prognosis (76). This indicated that telomere attrition-induced cellular senescence is closely associated with IPF pathogenesis. Previously, we have detected senescence-associated β-galactosidase (SA-β-Gal), a representative marker of cellular senescence, in the lungs of patients with IPF. Epithelial cells covering fibroblastic foci and cuboidal metaplasia in the active fibrosing area are mainly stained with SA-β-Gal, whereas no positive senescent cells are detected in the healthy lung (77), which further indicated that cellular senescence is at least partly involved in the pathogenesis of lung fibrosis in IPF.

The p16^INK4A^ expression levels in the lungs are positively correlated with the severity of pulmonary dysfunctions in patients with IPF (78). The removal of p16^INK4A^-positive cells mitigates the bleomycin-induced deterioration of pulmonary function without markedly altering lung fibrosis in *INK-ATTAC* mice. A similar benefit was achieved by administering D + Q (78). One pilot clinical trial examined the safety and efficacy of administering D + Q thrice a week for 3 weeks in patients with IPF, and D + Q improved performances in physical tests, such as 6-minute walk distance, 4-meter gait speed, and chair stand time (79). Other senotherapeutics examined for IPF include NADPH oxidase (NOX) 1/4 dual inhibitors. An imbalance between the levels of NOX4 and nuclear factor-erythroid 2-related factor 2 (NRF2) promotes the pathogenesis of lung fibrosis by enhancing cellular senescence in myofibroblasts, resulting in resistance to apoptosis and persistent fibrosis during IPF pathogenesis (80, 81). The NOX1/4 dual inhibitor GKT137831 attenuates bleomycin-induced lung fibrosis development in an aged mouse model (82). A phase II trial with GKT137831 involving patients with IPF is currently ongoing (NCT03865927).

Other anti-aging therapies, including rapamycin, nicotinamide riboside, nicotinamide mononucleotide, sirtuin activators are widely implicated in the treatment of COPD and IPF, and those anti-aging therapies are well summarized in recent reviews (3, 71). In the following sections, we summarize and focus on the therapeutic mechanisms and effects of representative drugs (metformin, statins, fibrates, and thiazolidinedione derivatives) against metabolic diseases on the pathogenesis of COPD and IPF.

### Metformin

Metformin, a biguanide anti-diabetic agent, has been used as a first-line drug for diabetes. The orally administered metformin is absorbed from the intestine and transported to the hepatocytes where it inhibits the mitochondrial respiratory chain complex I (83, 84). Subsequently, the intracellular adenosine triphosphate (ATP)/adenosine monophosphate (AMP) ratio decreases, resulting in the activation of AMP-activated protein kinase (AMPK). Activated AMPK suppresses gluconeogenesis in the liver and downregulates serum glucose levels (85). In addition to its anti-diabetic activity, several studies have demonstrated the senomorphic effect of metformin. The mechanism underlying the senomorphic effect of metformin has not been completely elucidated and is assumed to be diverse (86, 87). Metformin attenuates IGF-1 signaling by decreasing the blood insulin levels, resulting in the inhibition of mTORC1 signaling, which is postulated to be a major systemic senomorphic mechanism (88–91). Additionally, metformin may exert a systemic senotherapeutic effect and directly exhibit senomorphic activity. Metformin imported into the cell *via* organic cationic transporter 1 exerts its senomorphic effect through several mechanisms (92). AMPK activation by metformin inhibits the downstream mTORC1 signaling, which is accompanied by the enhancement of nutrition sensing and autophagy. Additionally, AMPK improves mitochondrial biogenesis by activating peroxisome proliferator-activated receptor γ coactivator-1α (92). Furthermore, AMPK contributes to epigenetic transcriptional regulation *via* histone modification and microRNA (92, 93). Metformin-mediated inhibition of mitochondrial complex I suppresses ROS with concomitant production of advanced glycation end-products, resulting in a decreased accumulation of macromolecular damage (94). Moreover, metformin downregulates the inflammatory cytokine levels *via* NF-κB inhibition, activates NRF2, and consequently mitigates SASP secretion (95, 96). Thus, metformin exerts its senomorphic effect through the above-mentioned pleiotropic mechanisms. Several studies have reported that metformin extends the lifespan of model organisms (95, 97–99).

The lifespan-extending effects of metformin have been reported in patients with diabetes (100–102). In addition to its contribution to reducing cardiovascular disease risk, observational studies have demonstrated that metformin treatment decreases the incidence of malignancy (103–106). Furthermore, metformin mitigates age-related cognitive function decline (107, 108). The Food and Drug Administration and National Institute of Health are conducting the Targeting Aging with Metformin study, which aims to demonstrate the effects of metformin in non-diabetic populations on various physiological and pathological conditions, such as cancer, dementia, cardiovascular disease, lifespan, and other age-related diseases (86). However, some groups argue that the therapeutic effect of metformin is not a universal phenomenon in non-diabetic patients and healthy elderly individuals (109–111).

Stimulation of cultured human bronchial epithelial cells (HBECs) with CSE promotes mitochondrial injury, resulting in a decreased oxygen consumption rate (OCR). Metformin concentration-dependently reverses the CSE-induced depletion of OCR (112). The same study also examined the effect of a metformin-containing diet on the COPD mouse model exposed to cigarette smoke for 6 months. Lung inflammation, emphysematous change, and airway remodeling in the metformin-containing diet-fed group were lower than those in the routine diet-fed control group. Additionally, oxidative stress, cell death, telomere attrition, and cellular senescence were suppressed in the metformin-containing diet-fed group (112). Other researchers have reported that the AMPK-activating drug adenosine analog 5-aminoimidazole-4-carboxamide riboside suppresses CSE-induced cellular senescence and SASP-related factors in BEAS-2B, a human bronchial epithelial cell line (113). Additionally, the metformin-containing diet prevented the progression of elastase-induced emphysematous changes in a mouse model (114). Furthermore, metformin activated the epithelial sodium ion channel ENaC in lung epithelial cells, which may be involved in COPD prevention (115). Previously, we reported that metformin attenuates CSE-induced cellular senescence by suppressing mTOR signaling through the upregulation of DEPTOR expression in HBECs (116). Thus, local lung administration may be sufficient for metformin to exert a senomorphic effect, especially under cigarette smoke exposure conditions.

Several clinical studies have reported favorable effects of metformin in patients with COPD. The clinical outcomes of drugs against metabolic diseases in patients with COPD and IPF are summarized in **Table 1**. The COPDGene study reported that in patients with COPD, lung function decline with age among metformin users is slower than that among non-metformin users (112). Another COPDGene analysis demonstrated that metformin reduced the frequency of disease exacerbations in patients with both COPD and asthma and that metformin use was associated with an improved respiratory symptom score (117). In contrast, another study revealed no apparent clinical benefit of metformin administration in patients with COPD who were hospitalized for disease exacerbation although this study evaluated a small number of patients for a short duration (118). A large retrospective analysis of patients with both COPD and type 2 diabetes reported that all-cause mortality in the metformin-treated group was lower than that in the matched control group (14). Furthermore, a recent study demonstrated that metformin prevents the decline in pulmonary diffusion capacity in patients with both COPD and diabetes (13).

**Table 1:** Clinical outcomes of drugs against metabolic disease in COPD and IPF.

	Disease	Clinical result	Study design	Ref.
**Metformin**	COPD	↓ progression of emphysema	retrospective	(112)
		↓ the incidence of exacerbation in patients with both COPD and asthma	retrospective	(117)
		No benefit for hospitalized patients with COPD exacerbation	RCT	(118)
		↓ all-cause mortality	retrospective	(14)
		Prevented the decline of lung diffusion capacity	retrospective	(13)
	IPF	↓ all-cause mortality and the incidence of hospitalization	retrospective	(119)
**Statin**	COPD	Prevented the decline of lung function	retrospective	(11)
		↓ the incidence of both COPD exacerbation and intubation	retrospective	(120)
		↓ COPD death	retrospective	(121)
		Prevented the decline of lung function	retrospective	(122)
		↓ all-cause mortality	retrospective	(123)
		No reduction in the incidence of COPD exacerbation (simvastatin)	RCT	(124)
		↓ the incidence of COPD exacerbation (simvastatin)	RCT	(125)
	IPF	↓ all-cause mortality. Improved 6-min walk distance	retrospective	(126)
		Prevented the decline of lung function	retrospective	(127)
**Fibrate**	COPD	↓ the risk of COPD	retrospective	(128)
**Thiazolidinedione derivative**	COPD	↓ the incidence of COPD exacerbation	retrospective	(129)
		↓ the incidence of COPD exacerbation	retrospective	(130)

↓: Reduced.

COPD, chronic obstructive pulmonary disease; IPF, idiopathic pulmonary fibrosis; RCT, randomized control trial.

Several studies have demonstrated the potential efficacy of metformin in treating IPF. We previously reported that metformin suppresses TGF-β-induced myofibroblast differentiation of lung fibroblasts (131). Metformin activates AMPK, which inhibits TGF-β-induced NOX4 expression and concomitantly enhanced ROS production in lung fibroblasts. In a bleomycin-induced mouse model, metformin attenuated NOX4 upregulation and SMAD phosphorylation, resulting in the amelioration of lung fibrosis development (131). This inhibitory effect of metformin on pulmonary fibrosis has been further confirmed by several groups with one study suggesting that metformin may reverse the established pulmonary fibrosis (132, 133). A clinical retrospective study of patients with both IPF and diabetes demonstrated that the all-cause mortality and hospitalization in the metformin-treated group were lower than those in the non-metformin-treated group (119).

The potential application of metformin in the treatment of aging-related respiratory diseases has been demonstrated in both COPD and IPF. However, the drug concentration used under *in vitro* conditions is higher than the maximum blood concentration (C_max_) required for clinical use. The clinical and experimental dosages of the drugs against metabolic diseases are summarized in **Table 2**. Clinical dose and clinical C_max_ were mainly based on the information from the National Library of Medicine in the USA, the electronic medicines compendium in the UK, and the Kyoto Encyclopedia of Genes and Genomes (KEGG) MEDICUS in Japan. The C_max_ level of metformin for clinical use is 6.2–12.1 μM, whereas the concentrations used under *in vitro* experimental conditions are in the range of 0.5–10 mM. Therefore, the previously elucidated *in vitro* anti-senescent mechanisms may not precisely explain the potential use of metformin in patients with COPD and IPF. Furthermore, the oral administration of metformin at a clinically available dosage may not be sufficient to obtain appropriate clinical benefits as a senotherapeutic agent. We speculate that inhalation therapy with a high local concentration may address this issue associated with the clinical application of these agents in the treatment of aging-associated respiratory diseases.

**Table 2:** Concentration of drugs against metabolic diseases in clinical and experimental setting.

	Clinical dose	Clinical Cmax (approximate)	*In vivo* experiment	Model	*In vitro* experiment	Cell type	Disease	Ref.
**Metformin**	850-2550 mg/day	6.2-12.1 µM	1% enriched chow	Mouse, smoking	0.25 nM-1M	Bronchial epithelial cell	COPD	(112)
			50-250 mg/kg, oral	Mouse, elastase			COPD	(114)
			2.5-5 mg/ml, in water	βENaC-Tg mouse	5 mM	Bronchial epithelial cell	COPD	(115)
					1-2 mM	Bronchial epithelial cell	COPD	(116)
			300 mg/kg, i.p.	Mouse, bleomycin	1-10 mM	Fibroblast	IPF	(131)
			65 mg/kg, i.p.	Mouse, bleomycin	0.5 mM	Fibroblast	IPF	(132)
			1.5 mg/ml, in water	Mouse, bleomycin	1-10 mM	Fibroblast	IPF	(133)
Statin								
Simvastatin	5-20 mg/day	5.0-9.6 nM	5 mg/kg, oral	Rat, smoking	0.1-10 mM	Vascular endothelial cell	COPD	(134)
Atorvastatin	10-40 mg/day	6.1-48.4 nM	20 mg/kg, i.p.	Mouse, bleomycin	10 µM	Fibroblast	IPF	(135)
			20-40 mg/kg oral	Rat, paraquat	3.6 µM-1.1mM	Alveolar epithelial cell	Lung injury	(136)
Pravastatin	20-40 mg/day	38.9-76.1 nM	3-30 mg/kg, i.p.	Mouse, LPS			Lung injury	(137)
Fibrate								
Ciprofibrate	100 mg/day	72.6-124.5 µM			150-600 µM	Fibroblast	IPF	(138)
			10 mg/kg, oral	Rat, smoking	2.5-80 µM	Airway smooth muscle cell	COPD	(139)
Fenofibrate	106.6-160 mg/day	24.9-32.7 µM			1-25 µM	Fibroblast	COPD	(140)
Gemfibrozil	1200 mg/day	59.9-99.9 µM	40 mg/kg, i.p.	Mouse, smoking	10 µM	Bronchial epithelial cell	COPD	(141)
Pemafibrate	0.2-0.4 mg/day	3.2-7.3 nM	0.5-2 mg/kg, i.p.	Mouse, bleomycin	10 µM	Fibroblast	IPF	(142)
Thiazolidinedione								
Rosiglitazone	4-8 mg/day	0.4-1.7 µM	6 mg/kg, oral; 10-300 µg/kg, i.n.	Mouse, smoking	0.1-3µM	Macrophage	COPD	(143)
Ciglitazone	N/A	N/A	3.5 µg/body twice a week, i.n.	Mouse, smoking	10 µM	Dendritic cell	COPD	(144)
			200-400 mg/kg, oral	Mouse, bleomycin	1-20 µM	Fibroblast	IPF	(145)
Troglitazone	N/A	N/A	200-400 mg/kg, oral	Mouse, bleomycin	1-20 µM	Fibroblast	IPF	(145)

Clinical dose and clinical C_max_ were mainly based on the information from the National Library of Medicine in the USA, the electronic medicines compendium (emc) in the UK, and the Kyoto Encyclopedia of Genes and Genomes (KEGG) MEDICUS in Japan. COPD, chronic obstructive pulmonary disease; IPF, idiopathic pulmonary fibrosis; i.p., intraperitoneal injection; i.n., intranasal administration; LPS, lipopolysaccharide; N/A, not applicable.

### Statins

Statins reduce cholesterol levels in hepatocytes by inhibiting the conversion of hydroxymethylglutaryl-CoA to mevalonate, which is the rate-limiting step in cholesterol biosynthesis in hepatocytes (146). The downregulation of cholesterol in hepatocytes promotes the nuclear translocation of sterol regulatory element-binding protein 2 (SREBP2) from the cytoplasm. In the nucleus, SREBP2 binds to the low-density lipoprotein (LDL) receptor-encoding gene promoter and upregulates the expression of LDL receptor. Subsequently, LDL in the serum is transferred to hepatocytes, leading to the downregulation of serum LDL levels (146, 147). In addition to cholesterol-lowering effects, statins exert other beneficial effects (also called pleiotropic effects) (148), and several studies have demonstrated the senotherapeutic property of statins. Hydrogen peroxide-induced cellular senescence is ameliorated by atorvastatin, pravastatin, and pitavastatin *in vitro* using human umbilical vein endothelial cells (149). Statin-mediated activation of Akt, through phosphorylation of Ser 473, led to expression of endothelial nitric oxide synthase, SIRT1, and catalase, all of which were implicated in the attenuation of cellular senescence in this study (149). In addition, fluvastatin inhibits the onset of endothelial progenitor cell senescence induced by *ex vivo* culture conditions, and its effect is independent of nitric oxide, ROS, and Rho kinase, but dependent on geranylgeranylpyrophosphate (150). Statins inhibit the prenylation of various proteins by blocking the formation of isoprenoid intermediates, which are essential for protein prenylation (151). This is postulated to be part of the mechanism for the suppression of cellular senescence because it leads to the upregulation of cell cycle-related proteins and downregulation of the expression of cell cycle inhibitor p27^Kip1^ (150). Another report showed that the administration of low-dose fluvastatin and valsartan increased the expression of longevity genes, including *SIRT1*, *PRKAA*, and *KL* in human subjects (152). Accordingly, statins act through various mechanisms to inhibit cellular senescence and have potential as senotherapeutic agents.

Large-scale retrospective studies conducted in several countries have reported the potential benefits of statins for patients with COPD in COPD-related hospitalization, cardiovascular complications, and mortality (11, 120–123). One study reported that statins may prevent the decline in pulmonary functions (11). Large-scale follow-up studies have been performed focusing on the role of statins in patients with COPD. The STATCOPE trial is a multicenter, placebo-controlled, randomized prospective study that focuses on the effect of statins on COPD exacerbation (124). In this trial, the primary outcome of the incidence of COPD exacerbation was not significantly different between the placebo and simvastatin-treated groups. Additionally, the period to first exacerbation was not significantly different between the placebo and simvastatin-treated groups, indicating no clinical benefit of simvastatin in preventing COPD exacerbation. However, this trial enrolled advanced high-risk patients with a decreased percentage of forced expiratory volume in one second and a history of emergency visits or hospitalization due to COPD exacerbation within one year, which may have affected the results. Although conclusive studies examining the benefit of statins in patients with mild to moderate COPD are not available, a recent study has attempted to clarify this question. A prospective double-blind study revealed that simvastatin significantly decreased COPD exacerbation (125). The beneficial effects of statins were also examined in COPD animal model. Oral administration of simvastatin ameliorated cigarette smoke-induced emphysema and pulmonary hypertension in rats exposed to cigarette smoke for 16 weeks, although the involvement of cellular senescence was not evaluated (134).

The therapeutic benefits of statins have also been reported in patients with IPF. Combined retrospective analysis of large-scale IPF trials for the anti-fibrotic drug pirfenidone (CAPACITY study and ASCEND study) revealed that statin users were associated with lower mortality and higher 6-minute walking distance than non-statin users (126). Additionally, overall hospitalizations, respiratory disease-related hospitalizations, and IPF-related deaths were low among statin users. A similar analysis was performed in another IPF trial for the anti-fibrotic drug nintedanib (INPULSIS study). Irrespective of nintedanib use, significant suppression of the decline in forced vital capacity/year was observed among statin users (127). The effects of statins were also examined in a bleomycin-induced lung fibrosis mouse model. Myofibroblast differentiation and pulmonary fibrosis in the group intraperitoneally administered with atorvastatin at a dose of 20 mg/kg bodyweight were attenuated when compared with those in the control group (135). Pravastatin attenuated lipopolysaccharide (LPS)-induced acute lung injury in mice (137), while atorvastatin inhibited paraquat-induced epithelial-mesenchymal transition (136).

Therefore, statins may alleviate the pathogenesis of both COPD and IPF through various mechanisms. However, their direct senotherapeutic effects on respiratory diseases are still unclear, and future studies are required to reveal the precise mechanism.

### Fibrates

Fibrates, which are peroxisome proliferator-activated receptor (PPAR) α agonists, are used for the clinical treatment of dyslipidemia (153, 154). PPARα is expressed mostly in the liver, adipocytes, and skeletal muscles although its expression is also detected in other organs and immune cells (155). Fibrates promote fatty acid metabolism by activating PPARα and its transcripts. Additionally, activated PPARα upregulates the expression of the lipid enzymes medium-chain acyl-coenzyme A dehydrogenase and long-chain acyl-coenzyme A dehydrogenase and regulates mitochondrial β-oxidation (156, 157). In addition to their effects on lipid metabolism, several studies have demonstrated the senotherapeutic potential of fibrates. Fenofibrate protects against aging-related renal damage and dysfunction by improving proteinuria, tissue remodeling, inflammation, and apoptosis through the activation of AMPK and SIRT1 signaling in aged mice (158). One study reported that fenofibrate decreased the accumulation of senescent cells and inhibited cartilage degradation by inducing apoptosis and autophagic flux (159). Fenofibrate-induced PPARα upregulation reverses the aging effect on monocytes as evidenced by the restoration of fatty acid oxidation accompanied by high levels of lipid droplet formation (160). PPARα downregulation is implicated in Paneth cell senescence in the intestinal epithelial niche (161).

Peroxisome biosynthesis and metabolism are markedly downregulated in the lungs of patients with IPF. Treatment with ciprofibrate or pemafibrate promotes peroxisome proliferation and downregulates myofibroblast differentiation of fibroblasts (138, 142). Additionally, fenofibrate suppresses TGF-β-induced myofibroblast differentiation independent of PPARα activation by downregulating mitochondrial respiration (140). Fibrates suppress neutrophil infiltration, increase vascular permeability, and promote inflammatory cytokine production in bleomycin-induced or LPS-induced lung injury models and reduce lung compliance (162–164).

Ciprofibrate suppresses cytokines involved in smoking-induced airway remodeling and smooth muscle hyperplasia (139). The induction of TFEB by gemfibrozil mitigates CSE-induced autophagy impairment in airway epithelial cells, resulting in the suppression of ROS production and cellular senescence (141). This suggests the potential efficacy of ciprofibrates in activating autophagy by modulating TFEB through PPARα activation during COPD pathogenesis. A population-based retrospective cohort study reported that in patients with dyslipidemia, the incidence of COPD development among fibrate users was lower than that among non-fibrate users within the 6-year observation period (128). These findings indicate that fibrate is a promising therapeutic for aging-related lung diseases and that it can function as a senotherapeutic by activating PPARα and its transcripts. In contrast to metformin, oral administration of fibrates at a clinical dosage may sufficiently achieve high drug concentrations to exert a senomorphic effect based on *in vitro* experiments (**Table 2**), suggesting that fibrates are potential candidates that can be repositioned as senotherapeutics.

### Thiazolidinedione derivatives

In addition to metformin, statins, and fibrates, thiazolidinedione derivatives (such as rosiglitazone and pioglitazone) have been reported to exert senotherapeutic effects. Rosiglitazone and pioglitazone function as PPARγ agonists and are used as oral hypoglycemic agents (165). Cellular senescence induced by angiotensin II is inhibited by pioglitazone in endothelial progenitor cells (166). In this study, pioglitazone prevents cellular senescence by downregulating angiotensin type 1 receptor. Pioglitazone also restores telomerase activity, which may also be involved in its anti-senescence activity. Age-related functional decline in renal mesenchymal stem cells contributes to the pathogenesis of chronic kidney disease. Indoxyl sulfate, a uremia-related toxin, evokes cellular senescence in renal mesenchymal stem cells, which is inhibited by pioglitazone (167). This effect of pioglitazone is, at least in part, attributable to the activation of PPARγ, which leads to suppression of prion protein gene expression. The senotherapeutic properties of thiazolidinediones have also been demonstrated in UV irradiation-induced cellular senescence in murine skin fibroblasts. Pioglitazone inhibits UV-induced cellular senescence through attenuating ROS production with concomitant suppression of cell cycle arrest-associated proteins such as p53 and p21 (168). The senotherapeutic potential of thiazolidinediones is also demonstrated using *in vivo* models. Pioglitazone ameliorates age-related renal dysfunction in 24-months old rats, which is attributed to increased klotho expression and decreased oxidative stress and mitochondrial injury (169). Long-term treatment with low-dose rosiglitazone extended the lifespan of aged mice (170). Additionally, rosiglitazone mitigated inflammation and tissue atrophy, improved cognitive ability, and alleviated depression-like symptoms (170). Further, patients with diabetes treated with pioglitazone had a lower mortality rate compared to non-PPARγ agonists users (170).

Several studies have demonstrated the efficacy of PPARγ agonists in treating respiratory diseases. A retrospective study on veterans with both diabetes and COPD revealed that the risk of COPD exacerbations in patients receiving PPARγ agonists was significantly lower than that in patients receiving other diabetes medications (129). Another retrospective study reported similar results with PPARγ agonists (130). The effects of other thiazolidinedione derivatives have been examined in mouse models. Rosiglitazone prevents the upregulation of neutrophil counts in the bronchoalveolar lavage fluid of mice exposed to cigarette smoke for a short duration (5 days) (143). Ciglitazone is reported to attenuate lung emphysema in mice chronically exposed to cigarette smoke (3–5 months) (144). The therapeutic efficacies of troglitazone and ciglitazone have also been demonstrated in the bleomycin-induced lung fibrosis mouse model (145).

Compared with those on other drugs, studies on senotherapeutic potential of thiazolidinedione derivatives and their application for treating aging-related respiratory diseases are at a nascent stage. Hence, further studies on thiazolidinedione derivatives are needed.

## Inhalation therapy

As we have described in this review, the drugs against metabolic diseases exhibit senotherapeutic properties and may be beneficial for the treatment of aging-related respiratory diseases. However, the usefulness and clinical application of these agents to respiratory disease remains uncertain, because of the high drug concentrations used in the experimental models compared to the expected drug concentrations in the lungs based on their approved oral doses. We speculate that a possible solution to these issues is the development of an inhalation treatment modality. Repurposing pre-existing drugs with potential efficacy for respiratory disease to suit inhalation therapy may save time and cost compared with developing a new drug from scratch. Currently, the clinically available inhaled drugs for respiratory diseases are mainly composed of inhaled steroids and bronchodilators. However, under experimental conditions, several drugs against metabolic diseases, such as statins and thiazolidinediones, have been investigated in asthma and pulmonary hypertension models. In comparison to the distribution during intraperitoneal or forced oral administration, better lung-localized drug distribution is demonstrated during inhalation and intratracheal administration of simvastatin in OVA-induced asthma mouse model (171). Simvastatin inhalation suppresses airway inflammation and remodeling in a dose-dependent manner. Intratracheal administration of pravastatin is shown to suppress bronchial goblet cell hyperplasia and reduce TNF-α and KC expression in bronchoalveolar lavage fluid, but not the expression of other chemokines or airway irritability in OVA-induced asthma mouse model (172). In a rat model of monocrotaline-induced pulmonary arterial hypertension (PAH), intratracheal administration of nanoparticulated pitavastatin attenuates the progression of PAH accompanied by a reduction in inflammation and pulmonary artery remodeling (173). In another study, the effects of rosiglitazone on PAH were examined. While no obvious effect of oral rosiglitazone administration on pulmonary hemodynamics is demonstrated, intratracheal administration of poly(lactic-co-glycolic) acid-based particles of rosiglitazone induces selective pulmonary vasodilation and reduces the proliferation of vascular endothelial cells and smooth muscle cells in rats with PAH (174). Furthermore, combined inhalation of rosiglitazone and sildenafil leads to improvement in cardiac function, delayed right heart remodeling, and inhibition of arterial muscularization in rats with PAH (175). Nebulized pioglitazone in combination with synthetic lung surfactant promotes lung maturation and attenuates the development of neonatal hyperoxia-induced lung injury (176). Interestingly, inhalation therapy using resveratrol as a senomorphic to treat respiratory disease shows that intratracheal administration preserves lung compliance and structure and prevents DNA damage in prematurely aging telomerase null (*terc*-/-) mice (177). In addition, resveratrol-β-cyclodextrin inclusion complexes significantly suppresses ZnCl_2_ smoke-induced acute lung injury through anti-inflammatory and anti-apoptotic mechanism (178).

Accordingly, we speculate that the development of inhalation therapy using drugs against metabolic diseases can be a promising approach for potential senotherapy; however, several concerns must be noted. High drug concentrations in the lungs during inhalation may evoke previously unrecognized toxicity. In addition, the conversion of the drug delivery system from oral to inhalation could cause critical pharmacological alterations based on drug properties and pharmacokinetics which should be carefully examined. Overcoming drug insolubility in water and low stability in solution should be an urgent task in inhalation therapy development. Furthermore, the development of efficient drug delivery devices is a critical problem. In future studies, these issues should be addressed for each drug before adopting them for clinical applications.

## Conclusions

Cellular metabolism is closely related to the mechanisms of cellular senescence. Hence, drugs against metabolic diseases may have senotherapeutic potential. Cellular senescence plays a pivotal role in the pathogenesis of various aging-related disorders. Hence, senotherapy can be a promising approach to develop efficient treatments for refractory aging-related respiratory diseases, including COPD and IPF. Drugs against metabolic diseases can be potentially repositioned as senotherapeutics. However, based on the experimental results, concentrations higher than those achieved by oral administration may be necessary to determine the clinical efficacy of some promising senotherapeutics in treating COPD and IPF. In addition to the oral administration modality, the development of an inhalation modality for the treatment of metabolic diseases can be an attractive approach to achieve high and effective local concentrations of drugs in the lungs without inducing systemic adverse events. Future studies should focus on determining appropriate drugs, optimal drug concentrations, and effective treatment modalities to develop clinically applicable therapeutics for aging-related respiratory diseases using drugs against metabolic diseases.

## Author contributions

SM, SI, and JA prepared the manuscript. All authors have made substantial contributions to the manuscript and have read and approved the final manuscript.
